# Proteinase K treatment improves RNA recovery from thyroid cells fixed with liquid-based cytology solution

**DOI:** 10.1186/s13104-018-3914-4

**Published:** 2018-11-20

**Authors:** Tomoo Jikuzono, Aya Horikawa, Tomoko Ishikawa, Mitsuyoshi Hirokawa, Iwao Sugitani, Takashi Inui, Osamu Ishibashi

**Affiliations:** 1 Laboratory of Biological Macromolecules, Department of Applied Life Sciences, Graduate School of Life & Environmental Sciences, Osaka Prefecture University, 1-1 Gakuen-cho, Sakai, 599-8531 Japan; 2 Department of Endocrine Surgery, Kanaji Thyroid Hospital, 1-5-6 Nakazato, Kita-ku, Tokyo, 114-0015 Japan; 3 Department of Endocrine Surgery, Nippon Medical School, 1-1-5 Sendagi, Bunkyo-ku, Tokyo, 113-8602 Japan; 4 Institute for Human Life Innovation, Ochanomizu University, 2-1-1 Otsuka, Bunkyo-ku, Tokyo, 112-8610 Japan; 5 Department of Diagnostic Pathology and Cytology, Kuma Hospital, 8-2-35 Shimoyamate-dori, Chuo-ku, Kobe, 650-0011 Japan

**Keywords:** Thyroid cancer, RNA, Liquid-based cytology, Fine-needle aspiration biopsy, RNA integrity number

## Abstract

**Objective:**

Fine-needle aspiration biopsy (FNAB), an important diagnostic tool given its simplicity, safety, and cost-effectiveness, is fast becoming a popular procedure in the diagnosis of thyroid diseases. Generally, cells isolated from biopsies are transferred directly to microscope slides to prepare smears for cytopathological examination; however, the technical difficulties of this procedure often cause poor reproducibility, which limits the accuracy of diagnostic results. Liquid-based cytology (LBC), in which isolated cells are collected in a fixative solution, is advantageous in that it facilitates the preparation of homogenous cytological specimens. However, LBC has not been applied to molecular diagnoses, such as RNA expression-based diagnosis, mainly because of difficulties in cell recovery and RNA isolation. This study was aimed to improve RNA extraction from papillary cancer-derived K1 cells and thyroid FNAB specimens suspended in LBC solutions.

**Results:**

K1 cells suspended in CytoRich-Red and CytoRich-Blue, fixatives for LBC, were efficiently recovered by trapping to glass-fiber filters. Importantly, subsequent Proteinase K treatment was essential for efficient RNA extraction from the fixed cells. This finding was also applicable to RNA extraction from CytoRich-Red-fixed thyroid FNAB specimens processed in the same way. Consistently, *U6* small nuclear RNA was detected in these RNA samples by reverse transcription-polymerase chain reaction.

**Electronic supplementary material:**

The online version of this article (10.1186/s13104-018-3914-4) contains supplementary material, which is available to authorized users.

## Introduction

Thyroid cancer is a malignant endocrine gland neoplasm accounting for approximately 3.4% of total cancer diseases (https://seer.cancer.gov/statfacts/html/thyro.html). The number of new cases of this cancer in 2017 is estimated to be 56,870 worldwide, ranking eleventh among all cancer types (https://seer.cancer.gov/statfacts/html/thyro.html).

The pathological diagnosis and classification of thyroid tumors are defined based on the recent World Health Organization classification system (http://www.pathologyoutlines.com/topic/thyroidwho.html). Thyroid nodules are observed in over 5% of the adult population and possibly give rise to both benign adenomas and malignant lesions; thus their occurrence is not a diagnostic sign of thyroid cancer (https://www.endocrineweb.com/conditions/thyroid/fine-needle-biopsy-thyroid-nodules). Hence, it is necessary to differentiate between benign nodules and cancerous solitary thyroid nodules at any rates. The thyroid has a great advantage over other tissues in that it is easily accessible to needles. Rather than removing a small piece of the tissue by operation (a conventional biopsy), thyroid cells can be removed by inserting a thin needle into the tissue and subjected to microscopic examination, a process called fine needle aspiration biopsy (FNAB). The cells collected for FNAB are sometimes treated with a liquid fixative containing alcohols and/or formaldehyde and subjected to cytological tests (liquid-based cytology; LBC). In general, different types of thyroid tumors can be easily differentiated by histopathological diagnosis. However, there are exceptions; for example, distinguishing between metastatic and non-metastatic minimally invasive (MI)-follicular thyroid carcinomas (FTCs) is currently difficult by any pathological modalities. Thus, it is necessary to establish a method for the molecular diagnosis of thyroid tumor to substitute or complement the histopathological method.

In recent years, RNAs that do not code proteins, i.e., non-coding RNAs (ncRNAs), have attracted wide attention in the medical sciences. MicroRNAs (miRNAs) are representative short (of approximately 22 nucleotides in length) ncRNAs involved in the post-transcriptional regulation of gene expression. To date, numerous studies have been conducted to identify dysregulated miRNAs, also called ‘onco-miRs’, in many types of cancer [1–8], including thyroid carcinoma [9–11]. Thus, onco-miRs have been considered possible biomarkers of cancers [12, 13]. As for thyroid cancers, we previously reported that *miR*-*221*, *miR*-*222*, *miR*-*10b* and *miR*-*92a* are highly upregulated in MI-FTC tissues and could therefore serve as diagnostic markers of MI-FTC [14]. However, LBC specimens have not been applied to miRNA analyses so far, mainly because of certain technical concerns. First, the rate of cell recovery from LBC specimens is generally low. Second, RNA extraction from cells processed with formaldehyde-containing fixatives is inefficient because intramolecular chemical crosslinks formed during cell fixation prevent RNA molecules from extraction [15].

In this study, our aim was to improve RNA recovery from human thyroid cancer cells processed with a fixative for LBC.

## Main text

### Materials and methods

#### Cell culture and fixation

Human thyroid papillary carcinoma-derived K1 cells were obtained from DS Pharma Biomedical Co. Ltd. (Osaka, Japan), and maintained according to the manufacturer’s instructions. Briefly, the cells were cultured in a mixture of Dulbecco’s Modified Eagle’s Medium (Wako, Osaka, Japan), Ham’s F12 nutrient mixture (Wako), and MCDB105 medium (Sigma-Aldrich, St. Louis, USA) (2:1:1) supplemented with 10% fetal bovine serum at 37 °C in an atmosphere of 5% CO_2_ and 95% air. These cells were suspended in 5 ml CytoRich-Red and/or CytoRich-Blue fixative solutions (Becton–Dickinson, Franklin Lakes, USA), commercial products developed for LBC, and stored at 4 °C for 3 days.

#### Thyroid FNAB specimens

Five papillary thyroid carcinoma cases were randomly selected for this study. We obtained written informed consent and approval for this study from the Ethics Committee of Kuma Hospital (local IRB number: 20130808-1). The aspirated thyroid cells in the needles were transferred to CytoRich-Red or CytoRich-Blue fixative solutions.

#### Cell recovery and RNA isolation

K1 cells and the thyroid FNAB specimens suspended in CytoRich fixative solutions were obtained by trapping using Whatman GF/C glass-fiber filters (GE-healthcare, Little Chalfont, UK) (Additional file 1: Figure S1A). The filters with the trapped cells were washed with PBS and incubated in 500 μl lysis buffer [10 mM Tris–HCl (pH 7.8), 5 mM EDTA and 0.5% SDS] with or without 400 mg/ml Proteinase K (Wako) at 55 °C. After the removal of filters, the cell lysis solution was mixed with 1.5-ml RNAiso Blood (Takara-bio, Kusatsu, Japan), and subjected to RNA isolation according to the manufacturer’s instructions. Isolated RNAs were dissolved in 11 μl RNase/DNase-free distilled water, and RNA concentrations were determined using Quanti Fluor RNA System (Promega, Madison, USA) and the Fluorescence Spectrophotometer F-7000 (Hitachi, Tokyo, Japan).

#### RNA quality assessment

The isolated RNA was analyzed using the Agilent 2100 Bioanalyzer (Agilent, Santa Clara, USA) in combination with the RNA 6000 Nano LabChip kit (Agilent), and its quality was assessed by RNA integrity number (RIN).

#### Real-time reverse-transcription PCR

For the quantitative reverse-transcription (RT)-PCR analysis of *U6* small nuclear RNA (*RNU6*) levels, TaqMan MicroRNA Assays (Thermo-Fischer, Waltham, USA) were used. Monitoring of *RNU6*-derived PCR products was performed on a DICE Real-Time PCR System (Takara-bio).

### Results and discussion

Application of LBC specimens to molecular diagnosis has been attempted in previous studies. Most of these studies, however, focus on genomic mutations, and studies on RNA analysis are limited. To our knowledge, the only study to evaluate RNA expression using FNAB specimens was conducted in medullary thyroid carcinomas [16]. In that study, the mRNA expression in leftover cells in needles was analyzed by conventional RT-PCR. Therein, the cells were directly put into cell lysis/denaturation solution, possibly leading to minimized RNA degradation and subsequent successful mRNA detection. However, it is difficult to perform this procedure routinely at a clinical site, mainly because of shortage of time and personnel. It would be convenient if residual LBC specimens could be used for RNA analysis after histopathological examination; however, this has not been achieved so far, possibly owing to the following reasons. Firstly, the cells in the alcohol-containing fixatives (CytoRich-Red; 23.3% isopropyl alcohol and 10% methanol, CytoRich-Blue; 44.0% ethanol and 5.0% methanol) are not efficiently recovered by simple centrifugation, since pelleted cells are rather crumbly, which complicates the removal of the supernatant. Another problem could be that RNA molecules are not efficiently recovered from the fixed cells, particularly those fixed with formaldehyde-containing fixatives that form intramolecular crosslinks, which prevent the release of RNA from insoluble cellular components.

**Fig. 1:**
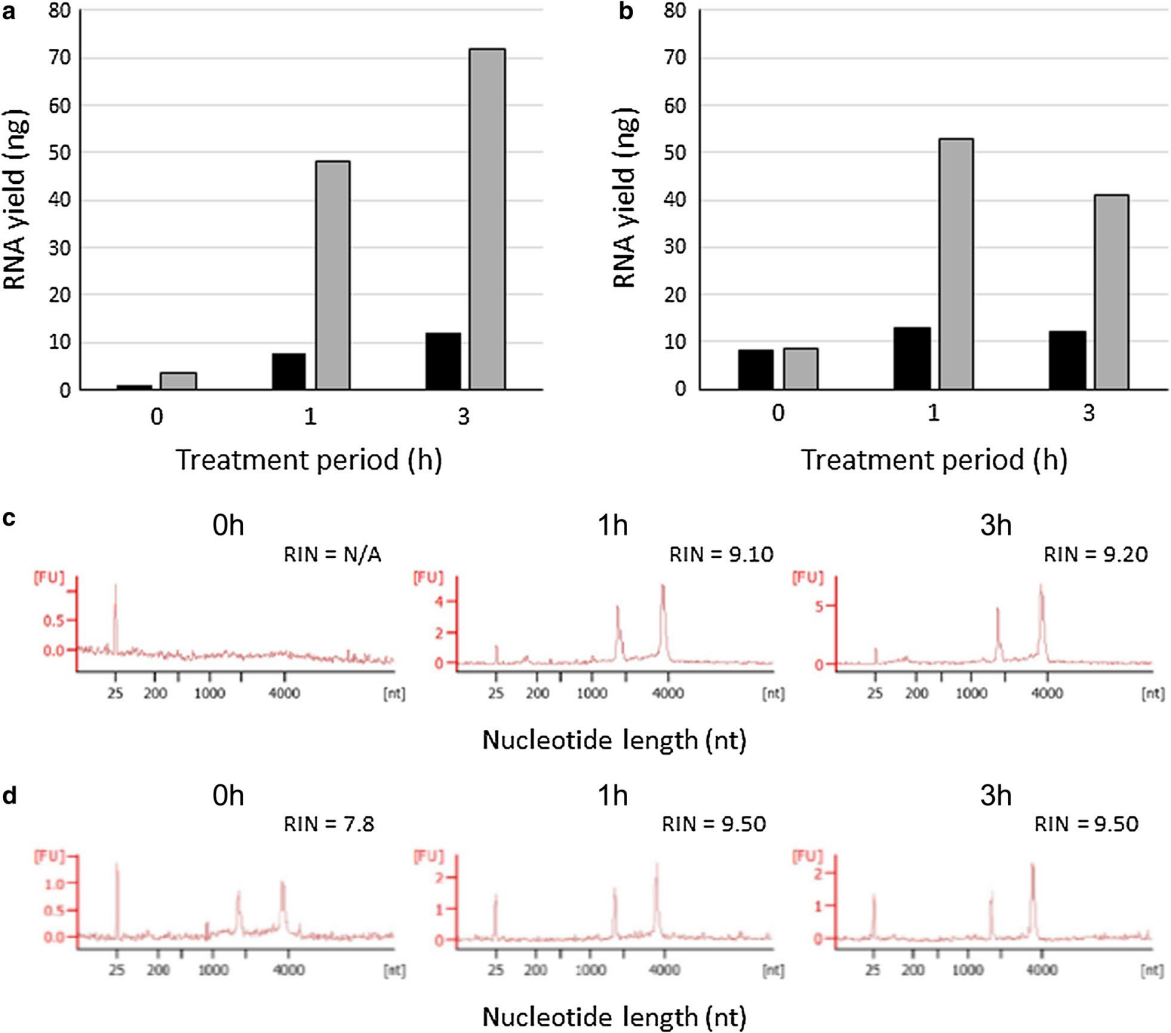
RNA recovery from filter-trapped K1 cells fixed for liquid-based cytology. RNA was extracted from the fixed K1 cells after incubation with Proteinase K-containing for the indicated periods at 55 °C. RNA recovery from K1 cells fixed with CytoRich-Red (**a**) and CytoRich-Blue (**b**). Black bars and gray bars represent the RNA yield from 20,000 to 100,000 cells, respectively. Quality of RNAs extracted from K1 cells fixed with CytoRich-Red (**c**) and CytoRich-Blue (**d**). The analysis using Agilent 2100 bioanalyzer revealed that 28S and 18S ribosomal RNAs (correspond to the bands at approximately 1900 nt and 3900 nt on the charts, respectively) remained nearly intact, leading to high RINs. *N/A* not assessed

To overcome the first problem, we tried to use a glass-fiber filter for cell tapping. The device for trapping cells in a glass-fiber filter is illustrated in Additional file 1: Figure S1A. After the K1 cell suspension was passed through the glass-fiber filter, only few cells were observed in the filtrate, indicating that the cells were efficiently trapped in the filter (Additional file 1: Figure S1B). Next, we evaluated the effect of Proteinase K treatment on RNA yields from the filter-trapped fixed K1 cells, because this treatment is known to improve RNA recovery from formalin-fixed paraffin-embedded specimens [14]. As shown in Fig. 1a, RNA yields were pronouncedly increased by treating the filter with CytoRich-Red-fixed K1 cells in a cell lysis buffer containing Proteinase K. Interestingly, this treatment was also effective on RNA recovery from K1 cells fixed with CytoRich-Blue (Fig. 1b), which does not contain formaldehyde. Thus, it is conceivable that Proteinase K treatment facilitates RNA recovery from rigid cells after dehydration. The results also show that RNA molecules were largely released from the fixed cells even after 1-h treatment. The RNA samples isolated from CytoRich-fixed cells were then subjected to quality assessment using Agilent 2100 Bioanalyzer. The RNA samples recovered from Proteinase K-treated fixed cells were shown to have an RIN of more than 9 (Fig. 1c, d), indicating that RNA integrity was well maintained during the procedure.

**Fig. 2:**
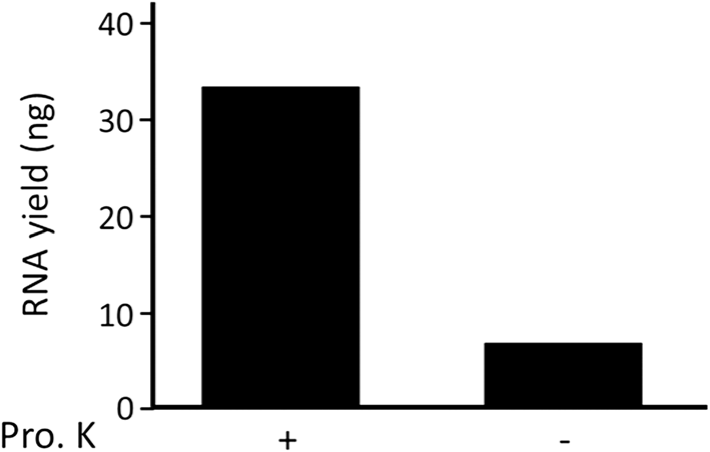
The effect of Proteinase K treatment on RNA recovery from CytoRichRed-fixed K1 cells. RNA was extracted from filter-trapped K1 cells after incubation with Proteinase K-containing and Proteinase K-free buffers for 3 h at 55 °C

**Fig. 3:**
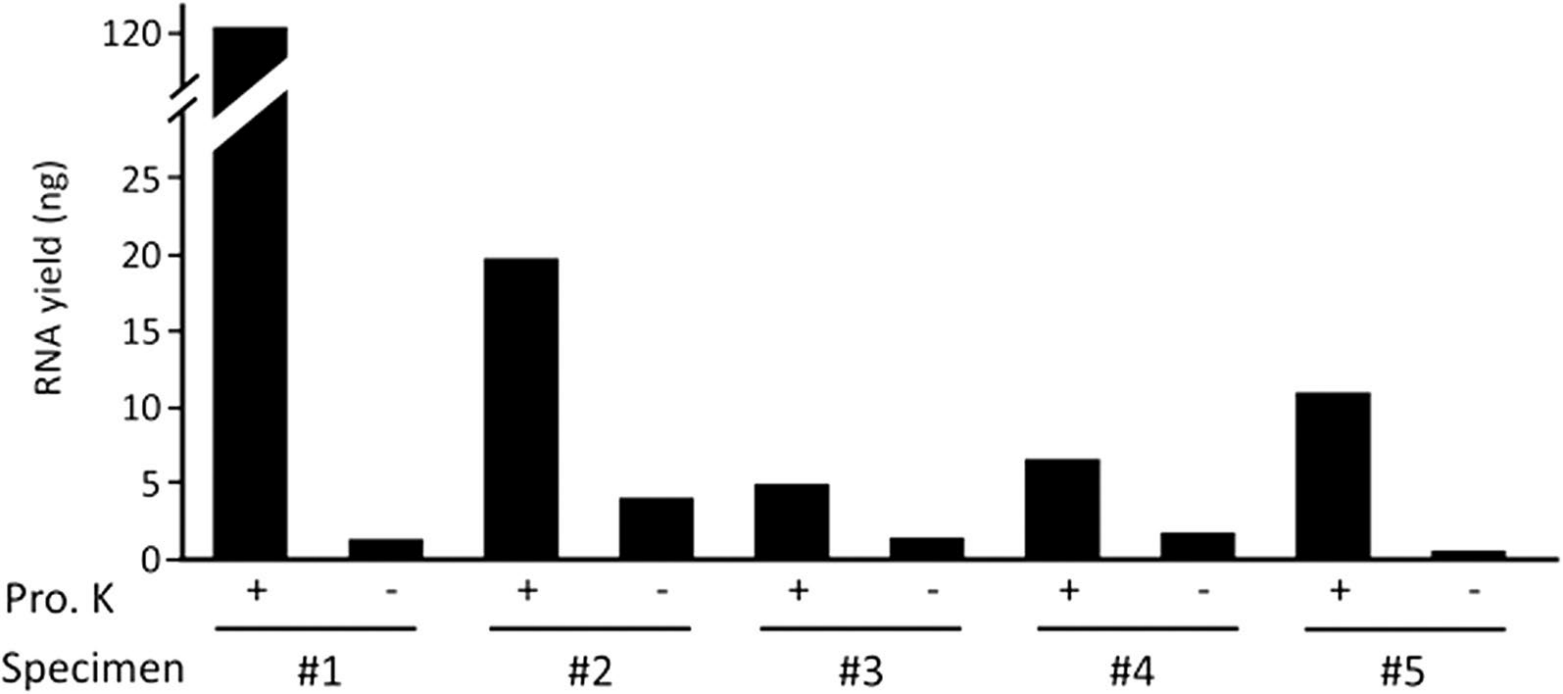
Effect of Proteinase K treatment on RNA recovery from CytoRichRed-fixed thyroid FNAB specimens. The thyroid FNAB specimens processed for LBC using CytoRich-Red solution were trapped to filters in the same way as described above. RNA was extracted from the specimens after incubation with Proteinase K-containing and Proteinase K-free buffers for 3 h at 55 °C

CytoRich-Red is more generally used than CytoRich-Blue for LBC of thyroid FNAB specimens, because CytoRich-Red efficiently lyses contaminated blood cells. Thus, only CytoRich-Red was used for the following experiments. To verify the importance of Proteinase K in RNA extraction, we extracted RNA from CytoRich-Red-fixed K1 cells incubated in both Proteinase K-containing and Proteinase K-free lysis buffers. As shown in Fig. 2, Proteinase K treatment pronouncedly improved RNA recovery from the sample, compared to poor recovery in the absence of Proteinase K. Then, we examined whether this finding is applicable to RNA extraction from clinical LBC samples. Five thyroid FNAB specimens fixed with CytoRich-Red solution were processed for RNA extraction in the presence or absence of Proteinase K. As expected, larger amounts of RNA were extracted from all these specimens in the presence of Proteinase K than in the absence of Proteinase K (Fig. 3).

To examine whether the isolated RNA was suitable for RNA-based molecular diagnostics, the RNA samples isolated from the CytoRich-Red-fixed K1 cells and thyroid FNAB specimens were subjected to quantitative RT-PCR to detect *RNU6* RNA, a ubiquitously expressed small nuclear RNA. As expected, *RNU6* RNA was detected in RNA extracted from the Proteinase K-treated materials (Additional file 1: Figure S2). The RNA samples extracted from the Proteinase K-untreated materials were not analyzed for *RNU6* expression as RNA yields were too low (Figs. 2, 3). Reaction without a cDNA template (negative control) gave no signal (Additional file 1: Figure S2).

Overall, our data suggest that RNA can be isolated efficiently from thyroid cells fixed with LBC solutions. LBC is also used in the diagnosis of other cancer types such as gynecological cancers [17]; thus, the method developed herein could be more widely applicable.

## Limitations

In this study we successfully isolated high-quality RNA from a tumor-derived cell line processed using a fixative generally used for LBC. We could also extracted RNA from clinical LBC specimens in the same way. However, we have not directly assessed the qualities of RNA extracted from clinical specimens as RNA yields are too low to measure RIN. Further, it is also necessary to examine the effect of fixation duration on RNA recovery.

## Additional file

**Additional file 1: Figure S1.** Cell trapping in a glass-fiber filter. (A) Scheme of cell trapping in a glass-fiber filter and RNA isolation. Cell suspension in a fixative was passed through the glass-fiber filter to trap the cells. The filter with trapped cells was subjected to RNA isolation. (B) Verification of cell trapping. Microscopic images of the cell suspension before and after filtration. After the K1 cell suspension was passed through the glass-fiber filter, only few cells were observed in the filtrate, indicating that the cells were efficiently trapped in the grass-fiber filter. **Figure S2.** Detection of *RNU6* RNA in RNA samples isolated from materials fixed with LBC solution. The total RNA (10 ng per reaction) isolated from the CytoRich-fixed K1 cells and thyroid FNAB specimens that were treated with Proteinase K was subjected to TaqMan-based quantitative RT-PCR to detect *RNU6* RNA. Assays were performed in triplicates. No amplification was observed when a cDNA template was omitted from the reaction (negative control).

## Abbreviations

FNAB: fine needle aspiration biopsy
LBC: liquid-based cytology
MI-FTC: minimally invasive-follicular thyroid carcinomas
miRNA: microRNA
ncRNA: non-coding RNA
RIN: RNA integrity number
RT-PCR: reverse transcription-polymerase chain reaction
