# Supplementary material for: Proteinase K treatment improves RNA recovery from thyroid cells fixed with liquid-based cytology solution
Source: BMC Res Notes. 2018 Nov 20;11:822. doi: 10.1186/s13104-018-3914-4 (PMC6245812; doi:10.1186/s13104-018-3914-4)
Supplement: Supplementary file 1 — Additional file 1: Figure S1. Cell trapping in a glass-fiber filter. (A) Scheme of cell trapping in a glass-fiber filter and RNA isolation. Cell suspension in a fixative was passed through the glass-fiber filter to trap the cells. The filter with trapped cells was subjected to RNA isolation. (B) Verification of cell trapping. Microscopic images of the cell suspension before and after filtration. After the K1 cell suspension was passed through the glass-fiber filter, only few cells were observed in the filtrate, indicating that the cells were efficiently trapped in the grass-fiber filter. Figure S2. Detection of RNU6 RNA in RNA samples isolated from materials fixed with LBC solution. The total RNA (10 ng per reaction) isolated from the CytoRich-fixed K1 cells and thyroid FNAB specimens that were treated with Proteinase K was subjected to TaqMan-based quantitative RT-PCR to detect RNU6 RNA. Assays were performed in triplicates. No amplification was observed when a cDNA template was omitted from the reaction (negative control). [file 13104_2018_3914_MOESM1_ESM.pdf]

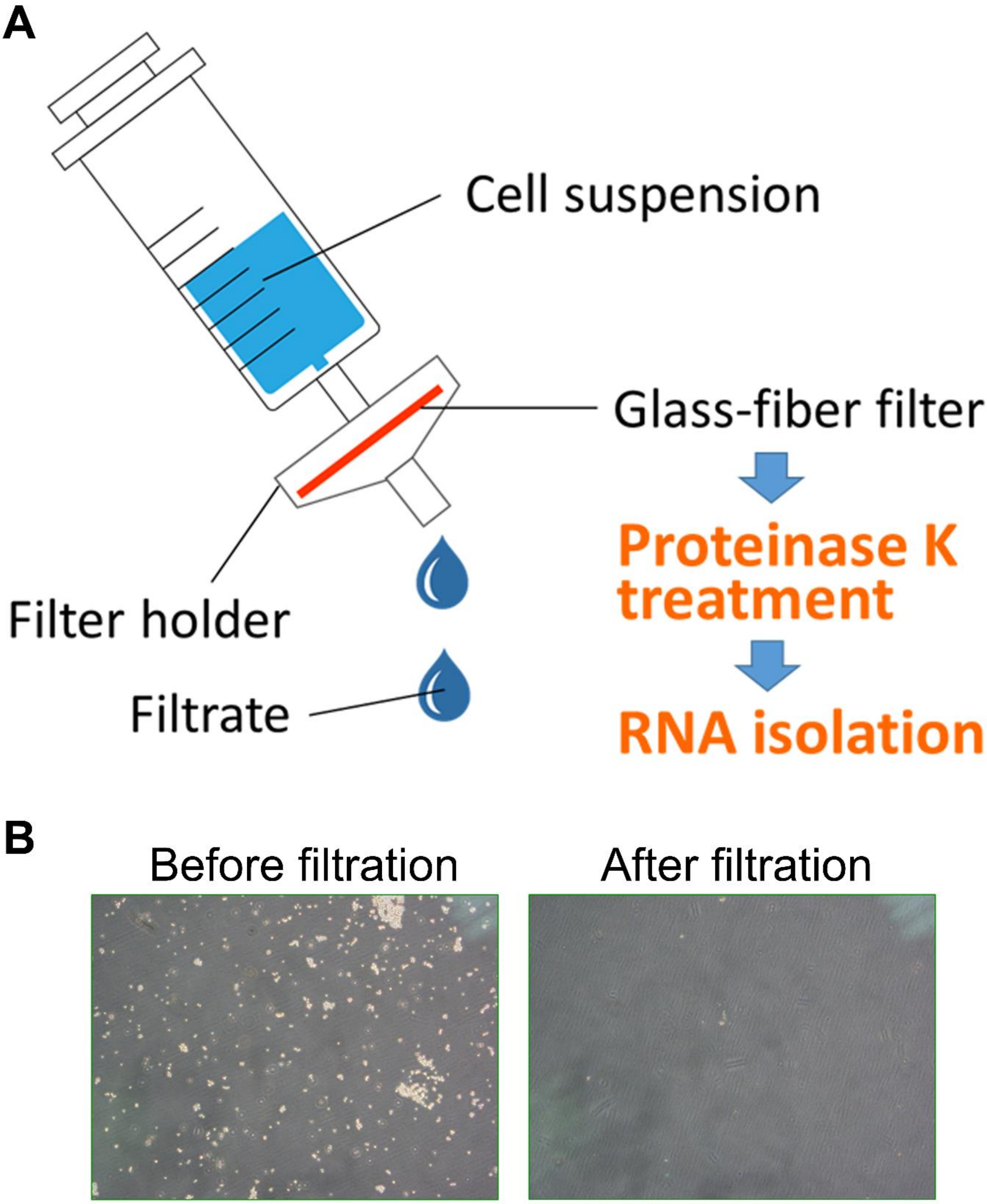

Fig. S1 Cell trapping in a glass-fiber filter
(A) Scheme of cell trapping in a glass-fiber filter and RNA isolation. Cell suspension in a fixative was passed through the glass-fiber filter to trap the cells. The filter with trapped cells was subjected to RNA isolation. (B) Verification of cell trapping. Microscopic images of the cell suspension before and after filtration. After the K1 cell suspension was passed through the glass-fiber filter, only few cells were observed in the filtrate, indicating that the cells were efficiently trapped in the grass-fiber filter.

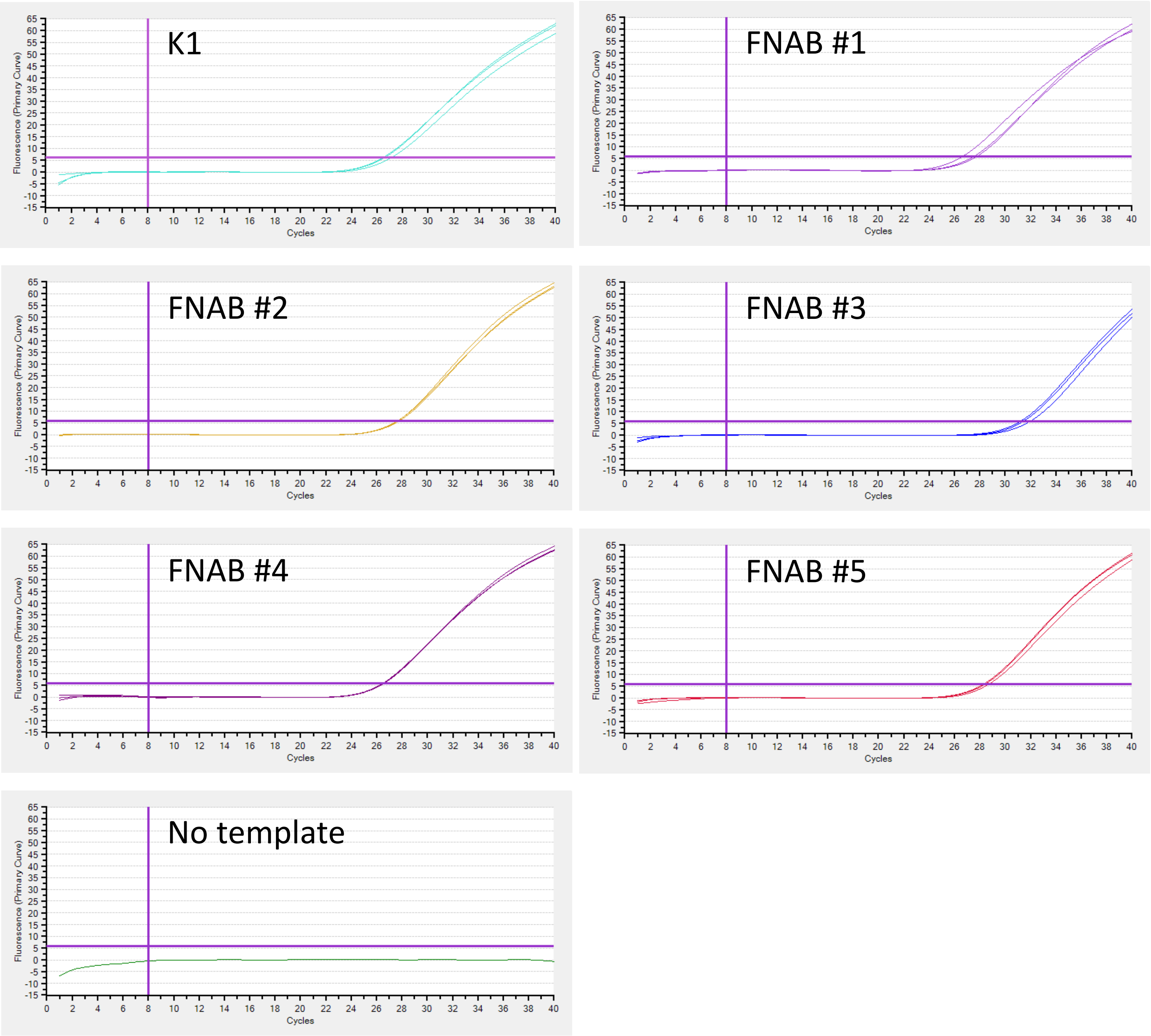

Fig. S2 Detection of *RNU6* RNA in RNA samples isolated from materials fixed with LBC solution

The total RNA (10 ng per reaction) isolated from the CytoRich-fixed K1 cells and thyroid FNAB specimens that were treated with Proteinase K was subjected to TaqMan-based quantitative RT-PCR to detect *RNU6* RNA. Assays were performed in triplicates. No amplification was observed when a cDNA template was omitted from the reaction (negative control).
